# Design and Fabrication of Mature Engineered Pre-Cardiac Tissue Utilizing 3D Bioprinting Technology and Enzymatically Crosslinking Hydrogel

**DOI:** 10.3390/ma15227928

**Published:** 2022-11-09

**Authors:** Shintaroh Iwanaga, Yuta Hamada, Yoshinari Tsukamoto, Kenichi Arai, Taketoshi Kurooka, Shinji Sakai, Makoto Nakamura

**Affiliations:** 1 Graduate School of Science and Engineering, University of Toyama, Toyama 930-8555, Japan; 2 Department of Clinical Biomaterial Applied Science, School of Medicine, University of Toyama, Toyama 930-0194, Japan; 3 Graduate School of Engineering Science, Osaka University, Osaka 560-8531, Japan

**Keywords:** inkjet bioprinting, biomaterial ink, enzymatic crosslinkable polymers, cardiomyocyte

## Abstract

The fabrication of mature engineered cardiac tissue is one of the major challenges in cardiac tissue engineering. For this purpose, we attempted to apply the 3D bioprinting approach. Aiming to construct an oriented tissue, a fine fiber-shaped scaffold with a support structure was first designed using CAD software. Then, a 3D bioprinter and cell-adhesive bio-inks were utilized to fabricate this structure. The cell-adhesive bio-inks were synthesized by combining sodium alginate and gelatin with tyramine, respectively, to form pre-gel materials that allow enzymatic crosslinking by horseradish peroxidase. By absorbance measurements, we confirmed that the tyramine modification rate of each polymer was 0.535 mmol/g-alginate and 0.219 mmol/g-gelatin. The width of the fiber-shaped scaffold was 216.8 ± 24.3 μm for the fabricated scaffold, while the design value was 200 μm. After 3D printing and adhesion-adding treatment of the scaffold with these bio-ink materials, cardiomyocytes were seeded and cultured. As a result, the cells spread onto the scaffold, and the entire pre-tissue contracted synchronously by day 6 of culture, showing a greater pulsatility than in the early days. Video analysis showed that the beating rate of pre-myocardial tissue on day 6 was 31 beats/min. In addition, we confirmed that the cardiomyocytes partially elongated along the long axis of the fiber-shaped scaffold in the pre-tissue cultured for 15 days by staining actin, suggesting the possibility of cell orientation. Furthermore, treatment with adrenaline resulted in a 7.7-fold increase in peak beating rate compared to that before treatment (from 6 beats/min to 46 beats/min), confirming the responsiveness of the pre-tissues to the drug. These results indicate that 3D bioprinting effectively produces mature cultured myocardial tissue that is oriented, contracts synchronously, and is responsive to drugs.

## 1. Introduction

Research on the fabrication of three-dimensional tissues by tissue engineering methods has been actively conducted to contribute to providing therapeutic tissues and organs and to the development of various fields of life sciences. While the realization of implantable, physiologically functional bioartificial tissues and organs will undoubtedly lead to destructive innovations in transplantation medicine, engineering advanced 3D tissues will also significantly contribute to medical research, bio-medicine, and drug discovery research [1]. Among them, tissue engineering of cardiac tissues is one of the key challenges in the field of tissue engineering, as well as a need of the hour, since cardiac diseases related to myocardial tissues are one of the leading causes of death worldwide. 

The heart is a very cell-dense muscular organ composed of several thick layers of the myocardium with oriented cardiomyocytes. Because the entire heart is composed of several overlapping layers of these oriented cardiomyocytes, the heart can contract effectively and pump blood efficiently when the cardiomyocytes contract in synchrony [2]. In most myocardial diseases, myocardial cells and these oriented tissue structures are simultaneously damaged and lost. Another essential feature of myocardial tissue is the abundance of capillaries in the three-dimensional myocardial tissue that provide sufficient oxygen and nutrients to all cells in the thick myocardial tissue.

There have been many attempts at cell transplantation with cardiomyocytes, mesenchymal stem cells, and cardiomyocytes from iPS cells to regenerate damaged cardiac muscle tissue. However, it remains challenging to restore the contractibility of myocardial tissue to the expected level only by transplanting single cells [3]. That is because cell survival, adhesion, and proliferation or differentiation at the transplanted site are poor. Furthermore, even if an effect is observed, it is considered to be due to the effect of paracrine secreted from cells. In addition, clinical trials of cell transplantation have been halted due to the side effects of arrhythmias. 

Many attempts have been made to fabricate engineered cardiac tissues by applying various tissue engineering approaches. Among them, bioprinting techniques have recently attracted attention as a method of tissue construction [4,5,6,7]. The bioprinting technique is a technique to place cells and materials at the target site in two or three dimensions. By doing so, it is expected to achieve a shape similar to that of living tissue and to create optimal environmental conditions by controlling the structure and composition of scaffolding materials. As a result, it is expected to improve the survival rate and function of cells within the tissue structure [8,9,10]. In research in cardiac tissue engineering, bioprinting technology has been used to create cardiac tissue models with complex vascular structures using patient-derived iPS cells [11], and the successful creation of heart valves that prevent backflow of blood was also reported [12].

The following important issue in this bioprinting approach is the post-fabrication process, which has been raised in the redefinition of biofabrication by the International Society for Biofabrication (ISBF), as well [13]. That is, how to mature the constructed tissue after the cells have been placed in their designed positions. Simply placing and culturing individual cardiomyocytes does not produce oriented cardiac tissue. Therefore, it is crucial to find a way to increase maturity and produce oriented myocardial tissue with high contractility. 

In this study, we aimed and challenged to engineer mature and oriented artificial myocardial tissue. We designed the structures that would be components of cardiac tissues, called bio-parts, and fabricated and provided them using 3D bioprinting technology and cell adhesive bio-inks. Myocardial cells were cultured on the scaffolds, and their maturity was investigated. In addition, the feasibility and usefulness of 3D bioprinting technology were discussed.

## 2. Materials and Methods

### 2.1. Designing a Scaffold for a Component of Cardiac Tissues

**Figure 1:**
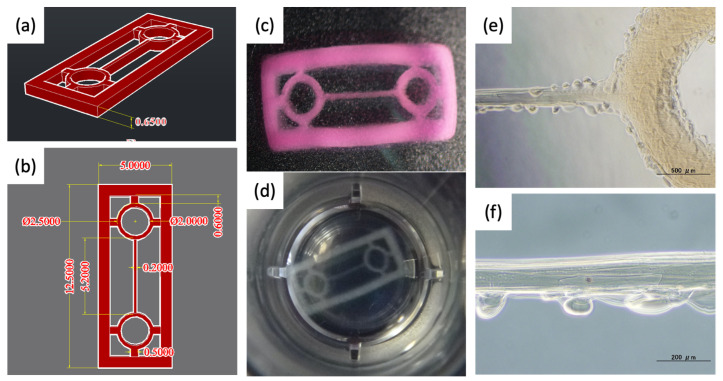
Design and photographs of acellular hydrogel scaffold for pre-cardiac tissues. (**a**,**b**) 3D images designed by AutoCAD (the unit of the values: mm). (**c**,**d**) Digital photographs of overview hydrogel scaffold. (**e**,**f**) Microscope images of the line part. Scale bar: (**e**) 500 μm and (**f**) 200 μm.

To fabricate oriented artificial myocardial tissue, we first designed thin fibrous structures as the shape of the constituent units of myocardial tissue. Culturing cells in an elongated structure would limit the direction of cell elongation to a specific way and promote orientation. Considering the nozzle diameter of our bioprinter, we designed the fiber portion of the scaffold as 200-μm width to ensure a certain cell adhesion area. However, such fine fibers are very fragile, so we designed the fibers with matching handling and structural support geometries at both ends (Figure 1a,b). By bundling and stacking such structures, oriented cardiac tissue may be assembled.

Using Autodesk’s AutoCAD 3D CAD software (Autodesk Inc., San Rafael, CA, USA), we designed a structure that integrates a thread-like main structure with 200-µm width, donut-shaped parts at both ends of the main structure for easy handling with tweezers and attachment to a support, and an outer frame part to support the overall structure. Finally, the data were converted to seven BMP format images for our 3D bioprinter.

### 2.2. Synthesis of Enzymatically Crosslinking Alginate and Gelatin

**Figure 2:**
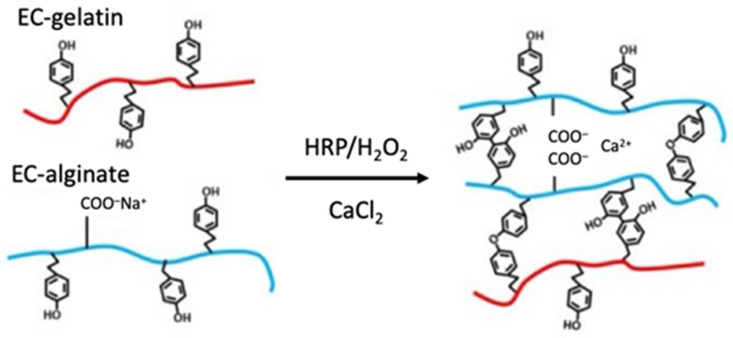
Schematic drawing of gelation of enzymatic crosslinkable polymers.

Enzymatically crosslinking (EC) polymers with phenolic moieties were synthesized according to the previously reported protocol [14]. Briefly, 0.5 g of sodium alginate (Kaigen Pharma Co., Ltd., Osaka, Japan), 0.913 g of water-soluble carbodiimide hydrochloride (WSC; Dojindo Laboratories, Kumamoto, Japan), 0.274 g of N-hydroxy succinimide (NHS; Sigma-Aldrich Co. LLC, St. Louis, MO, USA), and 1.03 g of tyramine hydrochloride (Tokyo Chemical Industry Co., Ltd., Tokyo, Japan) were dissolved in 100 mL of 0.1 mol/L MES buffer (2-morpholinoethanesulfonic acid; Dojindo Laboratories) and reacted for 24 h. After the reaction, the solution was dialyzed against ultrapure water and freeze-dried to collect EC alginate. In another batch, 2 g of gelatin (FUJIFILM Wako Pure Chemical Co., Ltd., Osaka, Japan) and 1 g of tyramine hydrochloride were completely dissolved in 100 mL of MES buffer at 60 °C, and the other chemicals (0.735 g of WSC and 0.221 g of NHS) were added to the solution. The reaction proceeded by stirring the solution for 24 h at 40 °C. EC gelatin was collected in the same way as EC alginate. The schematic image of the crosslinking reaction of the polymers by the enzyme is shown in Figure 2.

The degree of modified tyramine in each polymer was measured using the UV absorption of tyramine at 275 nm. EC polymers were dissolved in ultrapure water to prepare the solutions of 0.1 *w*/*v*%. The absorbance of these solutions was measured at 275 nm, and the following equation determined the degree of modified tyramine.

$$\mathrm{Degree}\;\mathrm{of}\;\mathrm{tyramine}\;\left(g/g-\mathrm{polymer}\right)=\frac{A_{275}\left(\mathrm{EC}\;\mathrm{polymer}\;\mathrm{of}\;0.1\%\right)-A_{275}\left(\mathrm{polymer}\;\mathrm{of}\;0.1\%\right)}{A_{275}\left(\mathrm{tyramine}\;\mathrm{of}\;0.1\%\right)-A_{275}\left(\mathrm{EC}\;\mathrm{polymer}\;\mathrm{of}\;0.1\%\right)}$$

### 2.3. Cell Adhesion Property onto EC Alginate Hydrogel Sheets Covered with EC Gelatin

**Figure 3:**
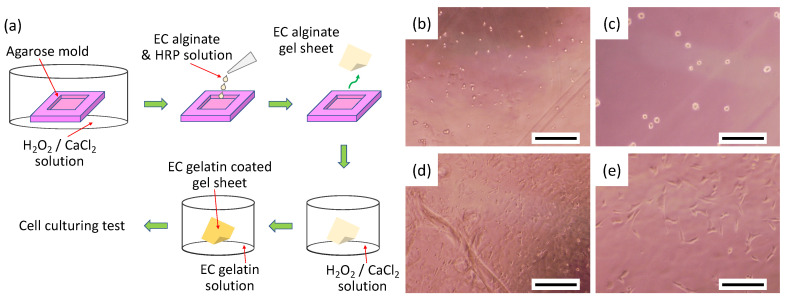
Cell adhesion onto EC alginate hydrogel sheet covered with EC gelatin. (**a**) Preparation of hydrogel sheet. Microscope images of cell adhesion onto EC alginate hydrogel (**b**,**c**) without EC gelatin coating and (**d**,**e**) with EC gelatin coating. Scale bar: (**b**,**d**) 500 μm and (**c**,**e**) 200 μm.

Agarose hydrogel molds were used to prepare flat EC alginate hydrogel sheets for evaluating cell attachment (Figure 3a). First, the agarose hydrogel with 200-μm thickness was cut to fabricate 1 cm square in length and width molds. These molds were immersed in a mixture of 2% CaCl_2_ and 5 mmol/L hydrogen peroxide solution (gelling solution) overnight at 4 °C. Next, a solution of 1.5% EC alginate and 50 units/mL of horseradish peroxidase (HRP) in saline was poured into the agarose mold. The gelation of EC alginate was performed chemically and physically within 30 min at room temperature. Next, EC alginate hydrogel sheets were moved into a 24-well plate and immersed with the gelling solution for 15 min at 4 °C. The gelling solution was aspirated, and 1% EC gelatin solution in saline was poured into each well. The EC alginate hydrogel sheets were washed twice with saline after a 30-min treatment of EC gelatin, then the surfaces of the alginate hydrogel were chemically modified with gelatin. Finally, these gelatin-covered alginate hydrogel sheets were immersed into Dulbecco’s modified Eagle medium (DMEM) supplemented with 10% fetal bovine serum (FBS), 100 units/mL penicillin, and 100 μg/mL streptomycin overnight. Subsequently, mouse smooth muscle cells (P53LMACO1; JCRB cell bank, Osaka, Japan) were seeded onto the hydrogel surfaces at 1.5 × 10^4^ cells/cm^2^ as a model cell and cultured for 24 h at 37 °C in a humidified atmosphere with 5% CO_2_. Cell attachment onto the hydrogel was observed under a phase-contrast microscope (CKX41, Olympus). FBS was purchased from biosera (Boussens, France) and other chemicals from FUJIFILM Wako Pure Chemical Co.

### 2.4. Fabrication and Pre-Treatment of Scaffolds Using a 3D Bioprinter

**Figure 4:**
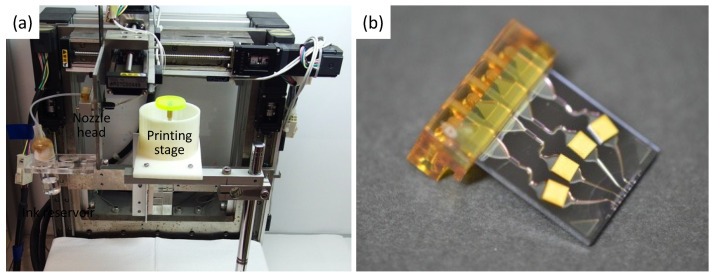
Our 3D inkjet bioprinting system. (**a**) Overview of the custom-made bioprinter and (**b**) nozzle head of piezoelectric type.

A custom-made 3D bioprinter developed in our laboratory was used to fabricate the designed scaffolds [15,16,17]. Our bioprinting system is adapted with a type of piezoelectric inkjet printer and is equipped with a mechanically controlled elevating stage, which allows the construction of elaborate 3D structures by adjusting the pitch width of the *z*-axis (Figure 4).

First, gel plates containing 5% agarose (FUJIFILM Wako Pure Chemical Co., Ltd.), 2% CaCl_2_, and 5 mmol/L hydrogen peroxide were prepared as a substrate for printing the scaffolds. Then, the designed scaffold structures were 3D-printed using a solution of 1.5% EC alginate and 50 units/mL HRP as biomaterial inks. By ejecting the biomaterial inks onto the agarose plates, EC alginate formed a hydrogel layer thanks to the diffusion of calcium ions, and the scaffolds were fabricated by stacked printing for seven layers of alginate hydrogel. The printing parameters of a 3D bioprinter are summarized in Table 1. The printed 3D hydrogel scaffolds were treated with EC gelatin to promote cell adhesion as in the previous method and stored at 4 °C in 2% CaCl_2_ solution until use.

### 2.5. Primary Culture of Neonatal Rat Cardiomyocytes

Animal experimental protocols were approved by the Animal Care and Use Committee of the University of Toyama (Approval number: A2015ENG-3 and A2018ENG-5) and conducted in accordance with the Institutional Animal Experiment Handling Rules of the University of Toyama. 

After 1-day old neonatal rats (Wistar; Sankyo Labo Service Co., Inc., Tokyo, Japan) were euthanized by decapitation, hearts were immediately removed, minced into 2 mm square pieces, and washed in Hanks’ balanced salt solution (HBSS) at room temperature. Minced heart tissues were dissociated using 150 units/mL-HBSS of collagenase type II solution in a conical flask shaking at 130 rpm at 37 °C. The dissociation process took 15 min per cycle, and four cycles were performed by replacing the collagenase solution. Cell suspensions from four dissociation processes were centrifuged at 200× *g* for 5 min. Isolated cells were resuspended in the culture medium containing 6% FBS, 40% Medium 199, 20 units/mL penicillin, 20 μg/mL streptomycin, 2.7 mmol/L glucose, and 54% balanced salt solution (BSS). The components of BSS for the medium are as follows: 116 mmol/L NaCl, 1.0 mmol/L NaH_2_PO_4_, 0.8 mmol/L MgSO_4_, 1.18 mmol/L KCl, 0.87 mmol/L CaCl_2_, and 26.2 mmol/L NaHCO_3_. The cell suspension was adjusted at a cell density of 7 × 10^5^ cells/mL in the culture medium and stored at 4 °C until seeding on the prepared scaffolds. All chemicals were purchased from FUJIFILM Wako Pure Chemical Co.

### 2.6. Fabrication and Observation of Engineered Cardiac Tissues

The prepared acellular scaffolds were placed on plastic sheets and set into a 24-well plate for ease of handling the scaffolds. In addition, the plastic sheets and the 24-well plate were treated with cell non-adhesive polymer solution (copolymer of butyl methacrylate and methacryloyl ethylbetaine, kindly provided by OSAKA ORGANIC CHEMICAL INDUSTRY Ltd., Osaka, Japan) to prevent cell attachment to these plastic materials. 

The myocardial cell suspension was plated at a cell density of 7 × 10^5^ cells/well and incubated at 37 °C in a humidified atmosphere with 5% CO_2_. The culture medium was replaced with a fresh one every 24 h.

Cell adhesion, elongation, and beating were observed in the fabricated myocardial tissues. The beating of the pre-cardiac tissues was captured in the movie under a microscope. The changes in the beating cycle with the number of culturing days were evaluated by image analysis of digital subtraction. The tones of the movies were briefly changed to grayscale by NIH ImageJ. Then, the displacement of cardiomyocytes at each time point was evaluated as a change in the brightness value by subtracting the image at time 0 from the movie images.

The orientation of cardiomyocytes was qualitatively assessed by staining their actin protein. First, the pre-cardiac tissues at 15-day culturing were fixed with 4% paraformaldehyde solution supplemented with 50 mmol/L CaCl_2_ overnight at 4 °C. Next, myocardial cells were permeabilized using 0.3% Triton-X in tris buffered saline (TBS, adjusted at pH 7.4) supplemented with 50 mmol/L CaCl_2_ at room temperature for 1 h. The cells were then stained with a mixture of Acti-stain 488 phalloidin (1:150 dilution; Cytoskeleton Inc., Denver, CO, USA) and Hoechst 33,342 (1:1000 dilution; Thermo Fisher Scientific Inc., Waltham, MA, USA) for 1 h at room temperature under dark conditions. Finally, the pre-cardiac tissues were rewashed, and fluorescent images of the cells were observed under a fluorescence microscope (BioZero, BZ-8000, Keyence, Osaka, Japan). 

### 2.7. Drug Testing and Ca Imaging of Pre-Cardiac Tissues

Pre-cardiac tissues on day 15 of culture were used to evaluate adrenergic responsiveness. First, they were transferred to glass-bottomed dishes and soaked in 1 mL of Fluoro Bright DMEM (Life Technologies Co., Carlsbad, CA, USA). The medium was then replaced with a loading buffer supplemented with 5 µmol/L of Fluo4-AM (Dojindo Laboratories) and 0.01 wt% of Pluronic F-127 (Sigma-Aldrich Co., LLC), and the pre-cardiac tissues were incubated for 20 min at 37 °C in a 5% CO_2_ environment. After incubation, the tissues were washed with Fluoro Bright DMEM three times and were observed under fluorescent microscopy. Subsequently, the medium was exchanged with Fluoro Bright DMEM containing 0.1 mmol/L of adrenaline (Bosmin injection, Daiichi Sankyo Co., Ltd., Tokyo, Japan), and the drug response of the tissues was observed. The obtained fluorescence changes were quantified by using NIH ImageJ.

## 3. Results and Discussion

### 3.1. Preparation of Biomaterial Inks

The absorbance at 275 nm was significantly higher for both EC alginate and EC gelatin than the original polymer (Table 2). This result indicated that we were able to introduce tyramine into each polymer. A calibration curve was prepared using an aqueous solution of tyramine chloride. The percentage of tyramine introduced into each EC polymer was calculated based on this curve, resulting in 0.535 mmol/g of tyramine introduction into alginate and 0.219 mmol/g into gelatin.

Sodium alginate is a polysaccharide composed of guluronic acid and mannuronic acid, and each of these constituent sugars has one carboxyl group. The unit mole number of monosaccharides is 5.05 mmol/g-alginate, which means that tyramine has been introduced into 10.6 out of 100 monosaccharide units in EC alginate. Since the sites of tyramine introduction are completely random in the synthesis using a condensing agent, the carboxyl groups derived from guluronic acid involved in calcium crosslinking of alginate [18,19] are also likely to be consumed for the modification. In general, it has been reported that modifications targeting the carboxyl groups of alginic acid decrease the gelation capacity of alginic acid as the modification rate increases [20,21]. Therefore, EC alginic acid will be similarly expected to decrease its gelation capacity.

On the other hand, using carboxyl groups in the modification would weaken intermolecular forces such as hydrogen bonding and electrostatic interactions of alginate molecules. As previously reported in our laboratory [16], we have found that the viscosity of EC alginate was reduced compared to unmodified alginate at the same concentration in an aqueous solution, suggesting that this is due to a decrease in electrical repulsion associated with a decrease in free carboxyl groups. We use the piezoelectric inkjet-type bioprinter in this paper. The ejective capacity of our bioprinter for the viscosity of biomaterial inks is about 10 mPa-s, making it difficult to print with high concentrations of alginic acid as ink. However, since EC alginate can be used as biomaterial ink in an aqueous solution with a higher concentration than unmodified alginate, the problem of reduced gelation capacity can be somewhat overcome by using a highly concentrated EC alginate solution. Although its modification per unit weight is less than half that of alginate, EC gelatin manifested a high modification rate compared to previous studies [22]. This result suggests that EC gelatin has an acceptable modification rate for chemical coating applications on the surface of EC alginate gels.

The results of cell seeding on gel sheets prepared from the resulting EC polymer are represented in Figure 2. The cells did not adhere and remained spherical on the surface of the EC alginate gel sheet without EC gelatin coating. In contrast, the cells attached and spread on the gel surface coated with EC gelatin. These results suggest that each polymer is sufficiently modified with tyramine. In our previous report, both EC alginate and EC gelatin were used in a mixed state as biomaterial inks to prepare the structures for tissues. However, while fibroblast cells were observed to extend when embedded in the mixed gel, no expansion of endothelial cells or cardiomyocytes could be observed. It is a known fact, especially for cardiomyocytes, that forming gap junctions with surrounding cardiomyocytes significantly affects beating [23,24]. Therefore, the construction of cell–cell interaction is essential for fabricating matured tissue. We can provide building blocks for tissue structures that promote synchronization of beating by seeding cells on the surface of hydrogel architectures.

### 3.2. Fabrication of Pre-Cardiac Tissues Utilizing Acellular Scaffolds

Figure 1 shows a scaffold design drawn by 3D CAD and a photograph of a hydrogel architecture fabricated by actually discharging EC alginate as a biomaterial ink using a 3D bioprinter based on the scaffold design. Regarding the macroscopic appearance, we confirmed that the shape was printed well, reflecting the design drawings. As expected, the construction of the circumferential structure facilitated handling with tweezers, and the outer frame increased the overall strength of the structure, resulting in the prevention of collapse of the structure after handling. 

On the other hand, observing the structure on a microscopic scale revealed ball-shaped bumps in some places. The size of these bumps was generally less than 100 μm. They were often found near the bottom of the structure rather than at the top, suggesting that gelation took a little time during the laminate printing stage. During that time, the ink droplets that had not yet become gelatinous dripped down and formed hydrogel in a different position than they should have. As for the reproducibility of the fiber structure construction, the CAD design value was 200 μm, whereas the measured value in the fabricated structure was 216.8 ± 24.3 μm (*n* = 5). Although some bumps were observed, our inkjet 3D bioprinting system succeeded in fabricating a structure that reflected the design with high accuracy.

The laminating print on the agarose hydrogel plates can easily collect and clean the printed architectures and observe the entire bodies. On the other hand, calcium ions and hydrogen peroxide, which are crosslinking agents, can only diffuse from the bottom agarose hydrogel substrate. Therefore, as the thickness of the structure increases, the ink layered on top will not form gels in time. As a result, the printed architecture is somewhat less detailed at the microscopic level.

**Figure 5:**
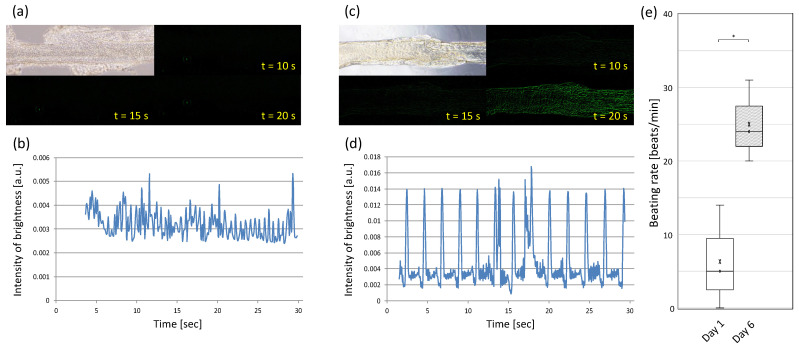
Analysis of cardiomyocytes beating on the line part of scaffolds. (**a**,**b**) Pre-cardiac tissues cultured for 1 day and (**c**,**d**) 6 days. (**e**) Beating rate on Day 1 and Day 6. *: *p* < 0.05. Each beating rate was calculated from video analysis (*n* = 3).

The results of seeding cardiomyocytes onto the acellular scaffold coated with EC gelatin are shown in Figure 5. The cardiomyocytes adhered and spread onto the surface of the fibrous part 1 day after seeding (Figure 5a). The cardiomyocytes had almost no beating on day 1, and the video analysis results showed little change in the brightness intensity due to their beating (Figure 5b). However, the pulsation was gradually observed after 2 days, and the entire structure was moving in a large periodic motion by day 6 of the culture (Figure 5d). The large periodic pulsation suggests that the myocardial cells on the scaffold may be connected and synchronized. Furthermore, as mentioned earlier, while it was not possible to observe the extension of cardiomyocytes or the overall beating of the cardiomyocytes inside the hydrogel made from the mixture of EC alginate and EC gelatin biomaterial ink, we succeeded in fabricating a dynamic beating pre-tissue by seeding cardiomyocytes on the scaffold of EC alginate covered with EC gelatin. These data indicated that preparing the cultural environment in a state suitable for each cell type is crucial to fabricating tissues closer to living organisms.

Bioprinting of myocardial tissue is challenging, and many studies have been conducted, but most of them have been performed by printing cells embedded in biomaterial inks. Although it is possible to promote cell–cell adhesion in gels by using materials more suitable for cell culture, such as collagen or decellularized ECM as ink, the formation of connections between cardiomyocytes on the surface of scaffolds, as we have carried out in this study, is somewhat deficient compared to seeding cardiomyocytes on the surface of scaffolds. However, compared to the method of seeding cardiomyocytes on the scaffold surface, as in the present study, there is concern that the formation of bonds between cardiomyocytes on the scaffold surface is somewhat impaired and that it may take longer for the cells to synchronize. In addition, since we are currently printing relatively small tissues, it does not take much time to fabricate structures, but if we were to print and model larger structures, it would take much longer to move to the culture process, which would be stressful for the cells. On the other hand, our method has the advantage of seeding cells after the structures are fabricated, so the cell post-culture period can be very short. Moreover, the preparation of many simultaneous, pulse-synchronized pre-tissues is expected to contribute to the fabrication of larger tissue structures by assembling them.

### 3.3. Cell Orientation onto the Fiber-Shaped Scaffold

**Figure 6:**
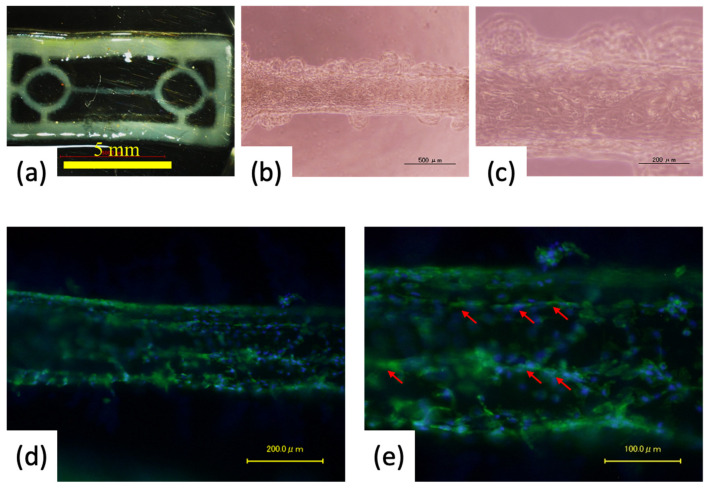
Evaluation of cell orientation of pre-cardio tissue cultured for 6 days. (**a**) Digital photo image of the whole tissue. (**b**,**c**) Microscopic images of cardiomyocytes spread onto the line area. (**d**,**e**) Fluorescent images obtained by phalloidin staining. Red arrows indicate oriented cells in the long-axis direction. Scale bar: (**a**) 5 mm, (**b**) 500 μm, (**c**,**d**) 200 μm and (**e**) 100 μm.

The pre-myocardial tissue on day 6 of the culture, shown in the video analysis results, appeared to beat significantly against the long axis of the fiber geometry. Therefore, we fixed the pre-tissue and evaluated the orientation appearance, albeit qualitatively, by staining actin present on the surface of the cells (Figure 6). The pre-myocardial tissue could be cultured for a long term without losing its shape significantly. Additionally, we observed that some cells extended along the long-axis direction onto the fiber-like portions. The designed width of the fiber-like portion was 200 µm, and as in the case of flat patterning culture, the area of the cells that could adhere and grow was limited compared to a large area such as a dish. In addition, the nozzle head was moved in a direction parallel to the long-axis direction of the fiber structure when printing the scaffold. That may have caused the scaffold to be incidentally printed with some alignment of the hydrogel direction in the long-axis direction of the fiber. Thus, it is presumed that the printing orientation may have influenced the printing of the hydrogel structure. We speculate that these factors may have caused some cells to become oriented.

We designed fibers with 200-µm width, fabricated them by 3D bioprinting, and created slightly oriented tissues, suggesting that bundling these fibers in the same direction may contribute to creating largely oriented tissues.

### 3.4. Evaluating Drug-Response of Cardiac Building Units

On day 15 of the culture, the entire pre-myocardial tissue was contracting synchronously. Focusing on the fiber portion, a similar synchronous contraction was observed. Fluorescence microscopy showed that the entire fiber section fluoresced with beating, and most of the myocardial cells were observed to be electrically synchronized. 

**Figure 7:**
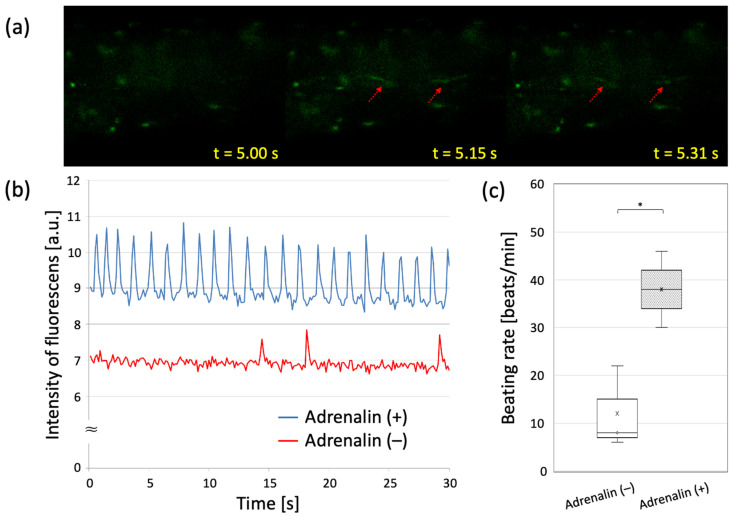
Drug responsiveness of pre-cardio tissue cultured for 15 days. (**a**) Fluorescent Ca imaging of the adrenalin-treated tissue. (**b**) Video analysis of cardiomyocytes beating before and after the addition of adrenalin. (**c**) Beating rate before and after adrenalin treatment. *: *p* < 0.05. Drug response studies were conducted with *n* = 3.

Figure 7 shows the results of the response to adrenaline administration in the precardiac tissue on day 15 of the culture. Changes in peak beating frequency were examined before and after the drug treatment. The untreated pre-tissue showed an average of 12 beats/min, while the treated pre-tissue averaged 38 beats/min. We confirmed that the pre-treated myocardial tissue responded to adrenaline with up to a 7.7-fold increase (6 beats/min to 46 beats/min) in beating rate.

It is known that cardiomyocytes in myocardial tissue are electrically coupled to each other by channel structures called gap junctions (GJs) [25,26,27]. Ions pass through these channels, and an excitatory stimulus initiated at one location in the tissue triggers the excitation of neighboring cell groups, spreading throughout the myocardial tissue. The formation of GJs is also essential for the propagation of excitation when culturing cardiomyocytes ex vivo. In the early stages of culture, the appearance of this gap junction is insufficient, excitatory propagation to neighboring myocytes does not occur, and synchrony is not observed. However, with the passage of several cultures, the pulses become synchronized. That is because the formation of gap junctions promotes extensive excitatory propagation and synchronization, reflecting maturation as a tissue [28].

In this experiment, Ca ion imaging confirmed the contraction and electrical synchronization of cardiomyocytes. Although we did not directly observe the connexins that form GJs this time, the observation of electrical synchrony suggests that GJs are being formed between cardiomyocytes. In the adrenaline loading test, the apparent increase in beating rate evidenced the drug responsiveness of the cells, and the concomitant synchronization of the beating rate increase response confirmed the maturity of the tissue as it progressed. The drug loading test effectively confirmed and assessed the maturity of individual cells and the tissue.

## 4. Summary and Conclusions

In conclusion, using the bioprinting approach, designed and engineered mature cardiomyocyte tissue was obtained, in which constituent cardiomyocytes were oriented, contracted strongly with electrical synchrony, and were responsive to adrenaline administration. The fiber-like scaffold design was thought effective in orienting the cells immediately. Moreover, inkjet bioprinting and cell adhesive EC hydrogels effectively produced the designed gel scaffolds. In addition, it was also confirmed that the constructed cardiomyocyte tissue could be made mature through the post-construction incubating process.

In the biofabrication approach, along with 3D bioprinting, the importance of bioassembly technology, which assembles tissue and organ parts mechanically, and the post-fabrication process, which advances the maturation process, has been advocated [13,29]. The engineered cardiac tissue successfully fabricated in this study was designed and produced as a component of bio-parts for bioassembly technology. Adding a culture process at this stage can produce bio-parts of myocardial tissue with an advanced maturation process. Furthermore, assembling these advanced maturation bio-parts will make it possible to produce more mature myocardial tissue.

## Figures and Tables

**Table 1:** The operating parameters of the 3D bioprinter.

XY-Axis Pitch Size [μm/pixles]	Z-Axis Pitch Width [μm]	Nozzle Head Speed [μm/s]	Number of Laminations
50	95	32,000	7

**Table 2:** UV absorbance and degree of tyramine of each polymer.

Chemicals	A275 [-] (at 0.1 *w*/*v*%)	Degree of Tyramine	
		[g/g-polymer]	[mmol/g-polymer]
Tyramine	8.63 *	—	—
Sodium alginate	0.002 ^(a)^	—	—
EC alginate	0.735 ± 0.001 ^(b)^	0.093	0.535
Gelatin	0.060 ± 0.005 ^(b)^	—	—
EC gelatin	0.374 ± 0.004 ^(b)^	0.038	0.219

* Calculated from calibration curve. (a) Standard deviation not shown due to extremely low value. (*n* = 3). (b) Data are expressed as mean standard deviation. (*n* = 3).

## Data Availability

Not applicable.
